# P-1546. Discordant Clinical and Microbiological Responses are Associated with Late Clinical Relapse in Uncomplicated Urinary Tract Infections: Pooled Analysis of EAGLE-2/EAGLE-3 Trial Data

**DOI:** 10.1093/ofid/ofae631.1713

**Published:** 2025-01-29

**Authors:** Amanda Sheets, Judith Absalon, Jeremy Dennison, Caroline R Perry, Nicole E Scangarella-Oman, Helen Millns, Salim Janmohamed

**Affiliations:** GSK, Collegeville, PA, USA, Collegeville, Pennsylvania; GSK, Collegeville, Pennsylvania; GSK, Brentford, UK, Brentford, England, United Kingdom; GSK, Collegeville, PA, USA, Collegeville, Pennsylvania; GlaxoSmithKline plc., Collegeville, Pennsylvania; GSK, Stevenage, UK, Stevenage, England, United Kingdom; GSK, Brentford, UK, Brentford, England, United Kingdom

## Abstract

**Background:**

Regulatory guidance recommends the primary endpoint for uncomplicated urinary tract infection (uUTI) trials be a composite of clinical and microbiological response. However, discordant outcomes have raised questions regarding the clinical utility of the microbiological component. The aim of this post hoc analysis was to explore the clinical significance of the microbiological endpoint based on pooled data from two recent Phase 3 trials in uUTI (EAGLE-2 [NCT04020341]/-3 [NCT04187144]).

**Figure:**
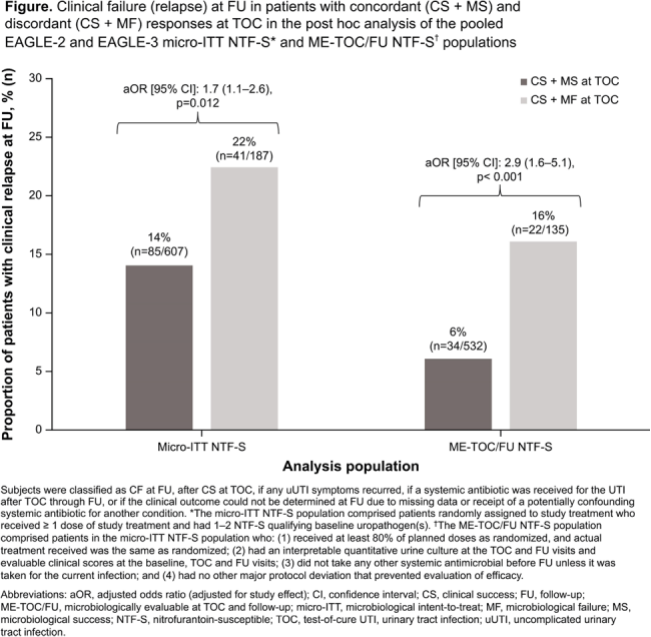

**Methods:**

EAGLE-2/-3 compared gepotidacin (1500mg) to nitrofurantoin (NTF; 100mg), both twice daily for 5 days in females aged ≥ 12 years with uUTI. Combined clinical success (CS) and microbiological success (MS) at test-of-cure (TOC) (days 10–13) was defined as quantified complete uUTI symptom resolution with no new symptoms and reduction of baseline qualifying uropathogens from ≥ 10^5^ to < 10^3^ CFU/mL, without other systemic antibiotic use. Pooled trial data with treatment groups combined were used to assess if discordant clinical and microbiologic responses at TOC impacted clinical responses at follow-up (FU; day 28±3).

**Results:**

In total, 1201 patients were included in the pooled population; 90% had *Escherichia coli* and 40% had history of recurrence. Overall, 22% of patients with discordant responses at TOC (CS + microbiological failure [MF]) became clinical failure (CF) at FU compared with 14% of patients with concordant success (CS + MS; **Figure**). The odds ratio of CF at FU for those with discordant vs concordant responses was 1.7 (95% confidence interval: 1.1–2.6, adjusted for study effect; p=0.012). Results were similar when the analysis was conducted excluding patients with missing data and protocol non-adherence (**Figure**).

**Conclusion:**

Discordant responses (CS + MF) at TOC were associated with increased risk of CF (relapse) at FU in this post hoc analysis. Based on the results of pooled EAGLE-2/-3 data, microbiological response appears to be an important component of treatment success criteria in uUTI trials and may predict clinical relapse.

**Funding:**

EAGLE-2 was funded in part by GSK and in part with Federal funds from the US Office of the Assistant Secretary for Preparedness and Response, Biomedical Advanced Research and Development Authority (HHSO100201300011C). EAGLE-3 was funded by GSK.

**Disclosures:**

**Amanda Sheets, PhD**, GSK: Employee|GSK: Stocks/Bonds (Public Company) **Judith Absalon, MD**, GSK: Employee|GSK: Stocks/Bonds (Public Company) **Jeremy Dennison, MD PhD**, GSK: Employee|GSK: Stocks/Bonds (Public Company) **Caroline R. Perry, PhD**, GSK: Employee|GSK: Stocks/Bonds (Public Company) **Nicole E. Scangarella-Oman, MS**, GSK: Employee|GSK: Stocks/Bonds (Public Company) **Helen Millns, PhD**, GSK: Employee|GSK: Stocks/Bonds (Public Company) **Salim Janmohamed, MD**, GSK: Employee|GSK: Stocks/Bonds (Public Company)

